# Subjective global assessment for nutritional screening and its impact on surgical outcomes: A prospective study in older patients with colorectal cancer

**DOI:** 10.1007/s00423-024-03548-w

**Published:** 2024-11-25

**Authors:** Fuminori Teraishi, Yusuke Yoshida, Ryohei Shoji, Nobuhiko Kanaya, Yuki Matsumi, Kunitoshi Shigeyasu, Yoshitaka Kondo, Shunsuke Kagawa, Rie Tamura, Yoshikazu Matsuoka, Hiroshi Morimatsu, Toshiharu Mitsuhashi, Toshiyoshi Fujiwara

**Affiliations:** 1 Department of Gastroenterological Surgery, Okayama University Graduate School of Medicine, Dentistry and Pharmaceutical Sciences, Okayama, 700-8558 Japan; 2 Department of Minimally Invasive Therapy Center, Okayama University Hospital, Okayama, Japan; 3 Perioperative Management Center, Okayama University Hospital, Okayama, Japan; 4 Center for Innovative Clinical Medicine, Okayama University Hospital, Okayama, Japan

**Keywords:** Subjective global assessment, Colorectal cancer, Older patients, Surgical outcome

## Abstract

**Purpose:**

Our perioperative management center provides preoperative intervention and functional and nutritional assessments for colorectal cancer patients aged over 75 years. This study evaluated the associations of preoperative nutritional status with postoperative outcomes and prognosis in colorectal cancer patients aged 75 years or older.

**Methods:**

This was a prospective, observational study of 71 colorectal cancer patients aged 75 years or older who underwent surgery between July 2020 and September 2022. The Subjective Global Assessment (SGA) was evaluated as a nutritional index. The patients were classified into three groups: SGA-A (well nourished), B (moderately malnourished), and C (severely malnourished), and the correlations with postoperative outcomes and prognosis were examined.

**Results:**

The median age of the 71 patients (34 males, 37 females) was 78 (75–92) years, and their median body mass index (BMI) was 22.3 (13.4–31.9) kg/m^2^. Forty-eight patients had colon cancer, and 23 had rectal cancer. On the SGA, 28 patients were SGA-A, 25 SGA-B, and 18 SGA-C. The SGA-B/C group had significantly higher BMI (*p* < 0.01) and more ICU admissions (*p* = 0.02). The G8 score was significantly lower (*p* = 0.03) in the SGA-B/C group, suggesting coexisting functional decline. In terms of postoperative outcomes, the SGA-B/C group had a significantly longer postoperative hospital stay (*p* = 0.04). The 3-year OS rates for all stages were 100% in the SGA-A group and 49.7% in the SGA-B/C group (*p* = 0.03), while the 3-year OS rates for patients excluding Stage IV were 100% in the SGA-A group and 68.5% in the SGA-B/C group, not significantly different (*p* = 0.14). The 3-year RFS rate was 95.5% in the SGA-A group and 65.3% in the SGA-B/C group (*p* = 0.15).

**Conclusion:**

The SGA is a promising nutritional index associated with short-term outcomes in older patients undergoing colorectal cancer surgery. The SGA can be assessed in a few minutes during an outpatient visit, making it useful for routine clinical use.

## Introduction

In cancer patients, the pathology of cancer cachexia caused by the inflammatory response of the cancer itself and the invasive procedures associated with cancer treatment such as surgery cause nutritional disorders, including decreased food intake and weight loss [1]. In the surgical treatment of cancer, preoperative nutritional disorders have been shown to have a significant impact on postoperative complications, postoperative recovery, and wound healing [2]. Older people are prone to nutritional disorders because they generally have decreased basal metabolism, decreased physical functions such as a decreased sense of taste and smell, poor chewing and swallowing, and decreased food intake, and they often have underlying diseases. In addition, psychological factors such as dementia and depression, and social factors such as living alone also become problems. Therefore, it is important to fully understand the characteristics of older cancer patients that may affect their nutritional status and to provide perioperative management tailored to their needs.

The subjective global assessment (SGA) is a widely used tool for evaluating the nutritional status of patients, including those with cancer [3]. It involves a comprehensive assessment of various factors related to nutrition, including weight change, dietary intake, gastrointestinal symptoms, functional capacity, and metabolic stress. The SGA has been reported to be a useful prognostic factor for patients with advanced colorectal cancer [4]. The purpose of this study was to evaluate the usefulness of the SGA as a nutritional screening tool, its impact on surgical outcomes, and its prognostic capability in older patients aged ≥ 75 years undergoing colorectal cancer surgery.

## Materials and methods

### Patients

This was a single-center, prospective, observational study conducted by the perioperative management center (PERiO), Okayama University Hospital. This cohort was managed in accordance with the ethical principles of the Declaration of Helsinki, and the protocol was approved by the ethics committee of our institution. We also followed the recommendations of the Equator Network and complied with the Strengthening the Reporting of Observational Studies in Epidemiology (STROBE) Statement: guidelines for reporting observational studies. Colorectal cancer surgery patients over 75 years of age in whom the PERiO had intervened between July 1, 2020, and September 30, 2022, excluding emergent surgery cases, were included. The following information was obtained from routine medical care. Basic patient characteristics, surgical findings, postoperative outcomes, and prognostic information were collected from electronic medical records. The data of comprehensive geriatric assessments were collected by PERiO nutritionists and nurses using the SGA and geriatric 8 (G8) during the preoperative interview.

### Nutritional evaluation, geriatric assessment, and outcome measurement

All patients included in the study underwent a consultation with a PERiO-affiliated dietitian prior to receiving treatment at our facility. During the nutritional consultation, the dietitian reviewed the SGA measures with the patients. The SGA is based on the information gathered from the history and physical examination, and the healthcare provider subjectively categorizes the patient into one of three nutritional status categories: SGA-A, well-nourished; SGA-B, moderately malnourished; or SGA-C, severely malnourished [3, 4]. The G8 is a screening tool to assess physical function, medications, nutrition, and mood in older people and is considered most useful because of its high sensitivity and acceptable specificity [5]. The primary outcome was the incidence of all postoperative complications according to the Clavien–Dindo classification [6, 7]. Complications were recorded for 30 days after surgery by the physician-in-charge. Secondary outcomes were length of postoperative hospital stay and the rate of direct discharge to home (for those patients admitted from home).

### Patient follow-up

In accordance with the guidelines of the Japanese Society of Colorectal Cancer [8], postoperative surveillance, including serum carcinoembryonic antigen levels (every 3 months), computed tomography (CT) (every 6 months), and colonoscopy (every 2 years), was usually performed for 3 years after surgery.

### Statistical analysis

Data were analyzed using IBM SPSS statistics (version 21; IBM Corp., Armonk, NY, USA), GraphPad Prism 6 (GraphPad Software, Boston, MA, USA), and Stata 17/MP (Stata Corporation, College Station, TX, USA). Continuous variables are described using medians and interquartile ranges (IQRs), and categorical variables are described using counts and percentages. Descriptive statistics were obtained for the incidence of postoperative complications, length of postoperative hospital stay, and discharge home rate. Correlations between patient information and observational and measurement items were also analyzed. The relationships between explanatory variables and outcomes were analyzed using logistic or linear regression. Logistic regression was used for binary outcomes, whereas linear regression was used for continuous variables. Two models were used to analyze the data: a crude analysis model (without adjustment for confounders) and an adjusted model (with adjustment for confounders). In the adjusted model, age, body mass index (BMI), operative time, and blood loss were included as confounding factors. These confounding variables were selected by the modified disjunctive cause criterion [9]. All statistical tests were two-tailed, with P < 0.05 taken to indicate a significant difference. Overall survival (OS) and relapse-free survival (RFS) were estimated using the Kaplan–Meier method, and comparisons were made using log-rank and Wilcoxon rank-sum tests.

## Results

### Patients’ characteristics and surgical outcomes

A summary of the patients’ characteristics and the results of the geriatric assessment are shown in Table 1. Data were obtained from 71 patients (34 men and 37 women) during the study period. Their median (IQR) age was 78 (75–92) years, and the median (IQR) BMI was 22.3 (13.4–31.9) kg/m^2^. Thirty-eight patients (53.5%) had primary history of abdominal surgery. Forty-eight patients had colon cancer, and 23 had rectal cancer. The median (IQR) serum albumin level was 3.9 (2.3–4.7) g/dl. On the SGA, 28 cases (39.4%) were SGA-A, 25 (35.2%) were SGA-B, and 18 (25.4%) were SGA-C. On the G8 score, 59 cases (83.1%) had 14 points or less. Minimally invasive surgery was performed in 69 (97.2%) patients, with a median (IQR) operative time of 230 (50–684) minutes and median (IQR) intraoperative blood loss of 5 (0–790) ml. Postoperative intensive care unit (ICU) management was required in 24 (33.8%) patients. ICU admission was often at the discretion of the anesthesiologist in charge. Fourteen patients with severe comorbidities were included, including 9 patients with severe cardiac diseases such as angina pectoris and valvular disease, 7 patients with cerebrovascular diseases, 5 patients with severe respiratory diseases, cirrhosis, chronic renal failure, and endocrine diseases.

**Table 1:** Baseline characteristics of 71 patients undergoing radical resection for CRC

Variables	
Age, yrs, median (IQR)	78 (75 − 92)
Gender, n (%)	
Male	34 (47.9)
Female	37 (52.1)
BMI, kg/m2, median (IQR)	22.3 (13.4 − 31.9)
History of abdominal surgery, n (%)	38 (53.5)
Tumor location, n (%)	
Colon	48 (67.6)
Rectum	23 (32.4)
Serum albumin, g/dl, median (IQR)	3.9 (2.3 − 4.7)
SGA score, n (%)	
A; well nourished	28 (39.4)
B; moderately malnourished	25 (35.2)
C; severely malnourished	18 (25.4)
G8 score, n (%)	
> 14	12 (16.9)
≦14	59 (83.1)
Minimum invasive surgery, n (%)	69 (97.2)
Operation time, min, median (IQR)	230 (50 − 684)
Blood loss, ml, median (IQR)	5 (0 − 790)
ICU admission, n (%)	24 (33.8)
pStage, n (%)	
0	2 (3)
I	17 (25.3)
II	13 (19.4)
III	29 (43.3)
IV	6 (9)
Chemotherapy, n (%)	20 (28.2)

*BMI* body mass index; *IQR* interquartile range; *pStage* pathological Stage

Perioperative results are shown in Table 2. Postoperative complications occurred in 15 patients (21.1%), including postoperative delirium in 8, ileus and suture failure in 2, and acute renal failure, urinary dysfunction, cholangitis, thromboembolism, chylous ascites, and unknown fever in 1 each. The median postoperative hospital stay was 9 (6–40) days, 64 patients were discharged directly to home (including institutionalized patients), and 7 patients were transferred to other hospitals, for a home discharge rate of 94.4%. The main reason for transfer was the lack of progress in postoperative weaning and the continuation of rehabilitation. Cases that required drain and stoma management were also included. There were no readmissions.

**Table 2:** Surgical outcome and types of postoperative complications

Variables	
Complications, C − D Grade I − V, n (%)	15 (21.1)
Type of complication	
Delirium	8
Ileus	2
Anastomotic leakage	2
Acute renal failure	1
Urinary dysfunction	1
Cholangitis	1
Thromboembolism	1
Chylous ascites	1
Unknown fever	1
Length of hospital stay, days, median (IQR)	9 (6 − 40)
Discharged directly to home*, n (%)	67 (94.4)
Readmission, n (%)	0 (0)

*C − D* Clavien-Dindo classification. *Admitted from private home only

### Correlations between SGA status and surgical outcomes

Table 3 summarizes the clinicopathological characteristics of the well-nourished SGA-A group (*n* = 28) and the malnourished SGA-B/C group (*n* = 43). Age, sex, history of abdominal surgery, primary tumor site, serum albumin level, minimally invasive surgery rate, operation time, intraoperative blood loss, pathologic stage, and postoperative chemotherapy rate were not significantly different between the two groups (*p* = 0.8, *p* = 0.84, *p* = 0.63, *p* = 0.76, *p* = 0.86, *p* = 0.07, *p* = 0.35, *p* = 0.56, respectively). Patients in the SGA-B/C group had significantly higher BMI (*p* < 0.01), more G8 scores ≤ 14 points (*p* = 0.03), and higher ICU admission rate (*p* = 0.02). On univariate analysis of the correlations between SGA status and postoperative outcomes (Table 4), postoperative complications and home discharge rates were not significantly different between the two groups (*p* = 0.59 and *p* = 0.83, respectively). When examining postoperative hospital stay, patients in the SGA-B/C group stayed significantly longer than those in the SGA-A group (11 days vs. 9 days, *p* = 0.04). (Table 5) shows the results of the multivariate analysis of the correlation between SGA variables and surgical outcomes, with results for the SGA-A/B and SGA-C groups that were significantly different in this analysis. In the adjusted model, the odds ratio for hospital transfer was 22.88 in the SGA-C group, and the rate of home discharge was significantly lower in the SGA-C group (*p* = 0.01), which was a significant predictor of hospital transfer. SGA status was not a significant predictor of postoperative complications.

**Table 3:** Associations of SGA status and clinicopathological factors in older patients with CRC

Variables	SGA A (*n* = 28)	SGA B/C (*n* = 43)	*p* value
Age, yrs, median (IQR)	78 (75 − 86)	78 (75 − 92)	0.8
Gender			0.84
Male	13	21	
Female	15	22	
BMI, kg/m2, median (IQR)	20.6 (13.4 − 31.6)	23.9 (17.1 − 31.9)	**0.01**
History of abdominal surgery, n (%)	14 (50%)	24 (55.8%)	0.63
Tumor location			0.63
Colon	18	30	
Rectum	10	13	
Serum albumin, g/dl, median (IQR)	4.0 (3.1 − 4.7)	3.9 (2.3 − 4.4)	0.24
G8			**0.03**
> 14, n (%)	8 (28.6)	4 (9.3)	
≦14, n (%)	20 (71.4)	39 (90.7)	
Minimum invasive surgery, n (%)	27 (96.4)	42 (97.7)	0.76
Operation time, min, median (IQR)	230 (102 − 422)	228 (50 − 684)	0.86
Blood loss, ml, median (IQR)	5 (0 − 340)	10 (0 − 790)	0.07
ICU admission, n (%)	5 (17.9)	19 (44.2)	**0.02**
pStage			0.35
0	2	0	
I	8	9	
II	5	8	
III	9	20	
IV	2	4	
Adjuvant chemotherapy in StageIII, n (%)	6 (66.7)	10 (50)	0.56

**Table 4:** Correlations of SGA status and postoperative outcomes in older patients with CRC. Results of univariate analyses examining the correlations between SGA status and postoperative outcomes

Variables	SGA-A (*n* = 28)	SGA-B/C (*n* = 43)	*p* value
Complications, C − D Grade I − V, n (%)	5 (17.9%)	10 (23.3%)	0.59
Length of hospital stay, days, median (IQR)	9 (6 − 29)	11 (6 − 40)	**0.04**
Discharged directly to home*, n (%)	25 (89.3%)	39 (90.7%)	0.83

**Table 5:** Results of multivariate analyses of SGA status and postoperative outcomes

Crude analysis model			Adjusted model						
Outcome	Variable	Odds ratio	95% confidencial interval		*p* value	Adjusted odds ratio	95% confidencial interval		*p* value
Hospital transfer	SGA C vs. A/B	4.76	0.95	23.82	0.06	**22.88**	**2.11**	**247.82**	**0.01**
Postoperative complication	SGA C vs. A/B	0.38	0.08	1.90	0.24	0.46	0.08	2.54	0.38
		Regression coefficient	95% confidencial interval		*p* value	Bias regression coefficient	95% confidencial interval		*p* value
Length of hospital stay	SGA C vs. A/B	2.80	−0.27	5.86	0.07	**3.46**	**0.41**	**6.50**	**0.03**

### Association between SGA status and prognosis

**Fig. 1:**
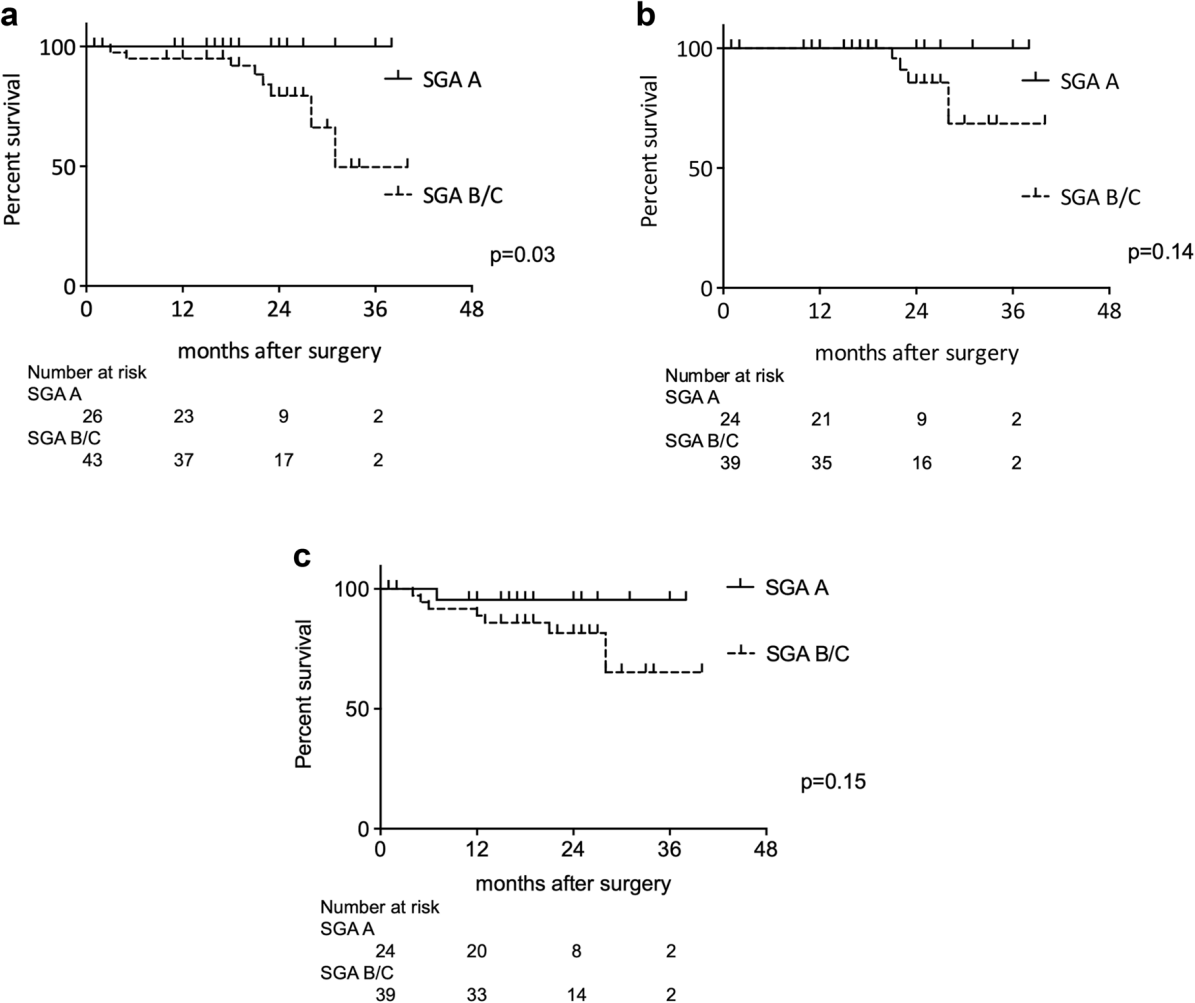
Kaplan–Meier survival curve analysis of overall survival (OS) and relapse-free survival (RFS) according to subjective global assessment status, **a** In patients in all stages, the SGA-B/C group had significantly worse OS than the SGA-A group (3-year OS of 49.7% vs 100%; *p* = 0.03). **b** In patients excluding Stage IV, OS was not significantly different between the SGA-A and SGA-B/C groups (3-year OS of 100% vs 68.5%; *p* = 0.14). **c** In patients excluding Stage IV, RFS was not significantly different between the SGA-A and SGA-B/C groups (3-year RFS of 95.5% vs 65.3%; *p* = 0.15).

Regarding the outcomes of all patients, 4 patients died of the primary disease, and 4 patients died of other diseases. Non-colorectal cancer-related deaths were due to other malignant diseases (*n* = 2), pneumonia (*n* = 1), and cerebrovascular disease (*n* = 1). There was one case of recurrence in the SGA-A group and five cases in the SGA-B/C group in patients who did not have Stage IV. The 3-year OS rates for all stages were 100% in the SGA-A group and 49.7% in the SGA-B/C group (*p* = 0.03) (Fig. 1a), while the 3-year OS rates for patients excluding Stage IV were 100% in the SGA-A group and 68.5% in the SGA-B/C group, not significantly different (*p* = 0.14) (Fig. 1b). The 3-year RFS rate was 95.5% in the SGA-A group and 65.3% in the SGA-B/C group (*p* = 0.15) (Fig. 1c).

## Discussion

In this study, 60% of colorectal cancer surgery patients aged 75 years or older were evaluated as malnourished by the SGA. The malnourished group was significantly more likely to have a G8 score of 14 or less, indicating functional decline, and more patients were admitted to the ICU and had a longer postoperative hospital stay. Furthermore, there was a significant independent correlation between SGA status and postoperative hospital stay after adjusting for age, BMI, operative time, and intraoperative blood loss. The findings of the present study are largely consistent with those of a previous small study that found a correlation between SGA and postoperative outcomes in patients undergoing colorectal cancer surgery [4]. BMI has been incorporated into various assessment methods as an indicator of nutritional assessment, but care must be taken when measuring height and weight in the elderly. There are problems with height measurement, such as underestimation due to the influence of turtleback and concerns about the accuracy of values measured in a non-standing position when the patient is unable to maintain a standing position. Also, weight measurement is often difficult for elderly persons with reduced ADLs, and they may rely on their own or family members' reports. Therefore, if height is measured with a shortened measurement or weight is over-reported, BMI values may be apparently increased [10], and it is possible that such measurement bias may have occurred in the SGA-B/C group with nutritional disorders in this study as well. In cancer treatment, preoperative nutritional disorders have been shown to affect postoperative complications, postoperative recovery, and wound healing, and to increase the incidence of infection, length of hospital stay, and mortality [2]. This is especially true in older patients. When oral intake is impaired due to postoperative complications after gastrointestinal cancer surgery, many older patients have difficulty in eating and recovering even after discharge from the hospital. Therefore, perioperative nutritional management should take these characteristics into consideration. Preoperative nutritional therapy has been reported to reduce the incidence of postoperative complications, shorten hospital stay, and improve prognosis [11, 12]. Since this was a prospective, observational study and almost all patients with SGA-B/C were able to take food orally, no special nutritional intervention was performed. Only one patient with obstructive symptoms and fasting management was given central intravenous nutrition preoperatively. We will continue to consider how to provide nutritional support for highly malnourished patients such as SGA-C.

There are several methods for assessing nutritional status, including BMI, prognostic nutritional index, skeletal muscle mass index, and SGA. Although these indices are related to cancer prognosis [13–16], the optimal cutoff values have not yet been elucidated; the SGA is a subjective method and has the major advantage of simplicity, not requiring any blood tests, and it can be performed in a few minutes at the initial visit. On the other hand, observer bias may affect SGA assessment because it relies on the ability of dietitians to collect and interpret data. In this study, the inter-rater reliability of different SGA users was not assessed. However, as we previously reported [17], the perioperative management at our institution was performed by PERiO, and SGA assessments were performed by well-trained dietitians with expertise and solid experience, minimizing the possibility of this bias. According to Neelemaat et al. [18], weight loss, decreased appetite, and use of nutritional supplements are three valid questions for nutritional screening in outpatients, making the SGA an ideal screening tool. Although the SGA is somewhat lacking in versatility because it is difficult to assess SGA in facilities where no dietitian is available, we assume that many facilities where surgery is performed have a dietitian on staff. In addition, SGA picks up patients with more obvious malnutrition, so early nutritional intervention may be difficult, but the time and manpower for nutritional care is relatively small, and it is only effective in hospitals with limited resources for nutritional cares. Recent reports have shown the usefulness of patient generated subjective global assessment (PG-SGA), a modification of SGA [19], and we plan to use PG-SGA in consultation with PERiO staff in the future.

The SGA is an index originally validated in surgical patients, and validation in other patient groups has focused on the association of SGA with other parameters and with postoperative outcomes in various cancer patients [4, 20–22]. In this study, we also examined the association between SGA and prognosis, and found a trend toward worse prognosis in the malnourished group (both OS and RFS), although the difference was not significant. This may be due in part to the lower rate of postoperative adjuvant chemotherapy in Stage III patients in the malnourished group (66.7% in SGA-A and 50% in SGA-B/C).

This study had several strengths and limitations. The main strength of this study was its prospective, observational design with continuous sampling. In addition, in the analysis of the correlations between SGA and postoperative outcomes, adjustment was made for confounding factors. It was found that age, BMI, operative time, and intraoperative blood loss were significantly associated with outcomes, and it was important to adjust for them. The first limitation of this study was the small number of cases. Although consecutive cases were enrolled during the study period, the sample size was relatively small (71 colorectal cancer patients) because this study included elective surgical cases aged 75 years or older. Due to the somewhat limited sample size, it was not possible to comprehensively assess predictors of OS and RFS. However, survival was not the primary endpoint of the study in the first place.

Second, the study included a variety of colorectal cancer procedures and failed to account for differences in surgical techniques. Nevertheless, there is some background similarity in the present study in terms of surgical invasiveness, since minimally invasive surgeries such as laparoscopic or robotic surgery were performed in more than 97% of cases. In addition, colon and rectal cancers were included in the same analysis because of their anatomic proximity and similar nutritional challenges. Third, preoperative nutritional intervention for patients evaluated as malnourished has not been standardized. Even in the malnourished group, many patients were able to take food orally and, with the exception of cases such as colorectal cancer ileus, they were eating normally at home until they were admitted to the hospital two days prior to surgery. In the future, preoperative nutritional intervention in selected cases should be tested to see if it improves postoperative outcomes. Furthermore, in order to validate the results of the current study, we would like to conduct a prospective, multicenter, observational study.

## Conclusion

In older colorectal cancer surgery patients, patients considered malnourished according to the SGA were more likely to have coexisting functional decline, which was associated with higher postoperative ICU admission rates and longer hospital stays also appeared to be affected. It is important to carefully consider preoperative nutritional status, as well as cancer stage, when developing strategies to improve outcomes for older colorectal cancer patients.

## Data Availability

No datasets were generated or analysed during the current study.
